# Macrophages in Healing Wounds: Paradoxes and Paradigms

**DOI:** 10.3390/ijms22020950

**Published:** 2021-01-19

**Authors:** Luisa A. DiPietro, Traci A. Wilgus, Timothy J. Koh

**Affiliations:** 1 College of Dentistry, University of Illinois at Chicago, 801 S. Paulina, Chicago, IL 60612, USA; 2 Department of Pathology, The Ohio State University, 129 Hamilton Hall, 1645 Neil Ave, Columbus, OH 43210, USA; traci.wilgus@osumc.edu; 3 College of Applied Health Sciences, University of Illinois at Chicago, 1919 W. Taylor, Chicago, IL 60612, USA; tjkoh@uic.edu

**Keywords:** macrophages, wound healing, inflammation, scar, regeneration

## Abstract

Macrophages are prominent cells in normally healing adult skin wounds, yet their exact functions and functional significance to healing outcomes remain enigmatic. Many functional attributes are ascribed to wound macrophages, including host defense and support of the proliferation of new tissue to replace that lost by injury. Indeed, the depletion of macrophages is unmistakably detrimental to normal skin healing in adult mammals. Yet in certain systems, dermal wounds seem to heal well with limited or even no functional macrophages, creating an apparent paradox regarding the function of this cell in wounds. Recent advances in our understanding of wound macrophage phenotypes, along with new information about cellular plasticity in wounds, may provide some explanation for the apparently contradictory findings and suggest new paradigms regarding macrophage function in wounds. Continued study of this remarkable cell is needed to develop effective therapeutic options to improve healing outcomes.

## 1. Introduction

In adult tissues, acute injury elicits a pronounced inflammatory response [1]. Following tissue damage, resident innate immune cells are activated, followed by the orderly infiltration of neutrophils and macrophages. Although the nuances of the timing and extent of inflammatory infiltration may vary slightly, the general pattern of inflammation is highly ordered in normally healing mammalian wounds. At first glance, the initiation of an innate inflammatory response in wounds seems an ideal reaction, as decontamination of the wound to prevent infection can be essential to survival. Yet decades of research now demonstrate that infiltrating immune cells do more in wounds than simply decontamination of microbes and removal of dying cells [2].

The concept that the acute inflammatory response might play a role beyond immune surveillance in wounds was first proposed about a century ago when Carrell demonstrated that white cells could produce “growth-promoting substances” [3]. Since that time, arguments for inflammation as either essential or dispensable to the repair process continue to be put forward [4]. On one hand, much is known about the critical role of macrophages in many types of wounds including skin, muscle, brain, and the peritoneal, pleural, and pericardial cavities, demonstrating that the innate immune response in tissue repair extends well beyond simple immune function [5,6,7,8,9,10]. In contrast, other studies suggest that certain non-infected wounds heal perfectly well with no or minimal acute inflammation [11,12,13,14]. As a versatile cell type that can adopt many phenotypes, macrophages have the capability to both destroy tissue and repair it [15]. In 2020, the thought pendulum regarding the role of macrophages in wound healing seems to have landed squarely on the side of “essential for repair”. Yet many enigmatic findings suggest that in certain wound healing and regeneration scenarios, macrophages may be dispensable. This review examines current concepts of the role and function of macrophages in healing wounds, with a focus on skin. We also contrast the role of macrophages in skin wounds with that described for tissues that fully regenerate following injury. An exploration of how and why macrophage function might be essential to the healing of certain skin wounds yet dispensable in other circumstances is provided. 

## 2. Macrophages in Wounds

### 2.1. A Brief Review of Macrophage Biology in Wounds

Many studies of macrophage function in healing wounds have utilized skin wound models. Easily accessible, and thus easy to study, skin provides an excellent prototype in which to study the complete wound healing response from start to finish. Macrophages in wounds come from two primary sources—resident cells and infiltrating cells derived from the circulation [16]. The exact time point of maximal macrophage content varies depending upon wound size and depth but most generally occurs just prior to full epithelial closure [17]. In normally healing wounds, macrophages decrease during wound resolution. In certain situations that impair wound resolution, such as the presence of foreign bodies, macrophages may persist. In such cases, macrophages may fuse to produce giant cells, which are large multi-nucleated cells that serve to prolong inflammation [18]. In most wounds, though, the macrophage content follows a predictable increase and decrease during the course of the repair process. 

Skin and indeed most tissues contain resident macrophages that can be quickly activated when injury occurs. However, many of the macrophages that are found in wounds are derived from circulating monocytes. Once attracted to the wound, monocytes differentiate into macrophages, allowing them to engage in multiple functional roles. Recently, macrophages have been shown to proliferate at sites of injury [19,20]; this proliferating population represents an important source of macrophages in wounds. Flow cytometry studies demonstrate that a specific population of less mature monocyte/macrophage cells enter a proliferative state of the cell cycle in wounds, while mature macrophages do not proliferate. Near the midpoint of healing, cells that are proliferating comprise up to 25% of this population. 

Macrophages within wounds are multi-functional cells with many possible phenotypes [21,22]. Early in the healing process, macrophages produce multiple cytokines and chemokines that stimulate the inflammatory response [23,24]. In this way, resident macrophages may provide critical signals that influence the infiltration of leukocytes and other cells into the injured tissue. Wound macrophages are actively phagocytic, clearing not just microbes but also dying cells and necrotic material [25]. The removal of apoptotic cells by macrophages, a process termed efferocytosis, is extremely well described for the removal of apoptotic neutrophils, a cell type that is abundant yet short-lived in early wounds. Multiple studies suggest that phagocytosis of senescent neutrophils by macrophages evokes a switch from a pro-inflammatory to a growth-promoting phenotype [26]. Macrophages probably clear many other types of apoptotic cells, such as dying endothelial cells and fibroblasts, suggesting that efferocytosis might be an important function of macrophages throughout the entire healing process. 

Beyond decontamination and cellular removal, the other well-studied role of wound macrophages is their ability to produce growth factors that stimulate tissue regrowth [9,27]. The capability of macrophages to produce growth factors, including proangiogenic and profibrotic molecules, has been shown both in vitro and in vivo in wounds [28,29]. Many therapeutic approaches to improving healing, including the addition of stem cells to wounds, have been suggested to depend upon shifting the macrophage phenotype to a growth-promoting one [30]. This growth-promoting activity is often believed to be essential to repair, an idea that will be further considered below. 

Given the complex functions of macrophages in wounds, heroic attempts to stratify wound macrophages into specific phenotypes have been made by several groups [21,22]. In general, macrophages in the early wound fall more into the pro-inflammatory or M1 phenotype, while those that persist into later phases exhibit a reparative or M2 phenotype, of which there are clearly many variants. In comparison to the classification of macrophage phenotypes in vitro, macrophages that are isolated from wounds often do not fall into neat categories [22,31]. Although trending towards M1 or M2, many wound macrophages exhibit both M1 and M2 markers. In short, wound macrophages span a wide range of complicated phenotypes that are neither completely M1 nor M2, a situation that has frustrated those who are eager to understand macrophage function in wounds. The origin of inflammatory versus reparative macrophages in wounds is not yet completely understood, although recent studies suggest that Cx3CR1^eGFP-hi^ reparative macrophages in skin wounds are derived from long-term tissue-resident macrophages, whereas cells expressing low-intermediate Cx3CR1^eGFP^, which are pro-inflammatory, are derived from the circulation [20]. 

In addition to inflammatory and reparative roles, some recent studies suggest that macrophages may assist in the resolution phase of healing. The final phase of wound healing, resolution, involves downregulation of inflammation, pruning of the capillary bed back to normal levels, maturation of the epithelium, and remodeling of the extracellular matrix towards a more normal architecture. Macrophages are well described to promote wound angiogenesis, stimulating and directing capillary growth [27,32]. Studies that are more recent suggest that macrophages can also support capillary removal and refinement of the vascular bed during wound resolution by producing anti-angiogenic factors and by phagocytosis of apoptotic endothelial cells [33,34]. The resolution phase of healing has not received much experimental attention, making this a potentially productive new area of investigation. While many questions about the spectrum of wound macrophage phenotypes are not yet answered, the advent of single-cell analysis should allow for a better of picture of the spectrum of wound macrophage phenotypes over the course of healing. 

### 2.2. Macrophages as Essential for Repair

Several extremely well validated studies have demonstrated the need for macrophages for proper repair of skin. The first of these was the landmark study of Leibovich and Ross, published more than 40 years ago [35]. In this study, treatment of guinea pigs with both systemic hydrocortisone and local injection of anti-macrophage serum was employed to deplete both circulating monocytes (the precursor to tissue-bound macrophages) and tissue macrophages. Wounds in animals with complete macrophage depletion showed impaired healing, including decreased wound debridement, lower numbers of fibroblasts, and decreased amounts of collagen. While the observations are compelling, they were primarily non-quantitative and the procedures used to deplete macrophages likely had several off-target effects. 

With the advent of new tools and improved methodologies, studies that are more recent have employed sophisticated approaches that circumvented concerns of off-target effects of the pharmacologic treatments used in the Leibovich study. Perhaps the most definitive evidence comes from three separate studies of skin wound healing published from 2009 to 2010 [6,7,36]. These studies stand out due to the use of sophisticated genetic approaches to eliminate monocytes and macrophages, along with the reproducibility of the findings across three different groups from two different continents. Each of these studies used murine strains with monocyte/macrophage-restricted expression of the human receptor for diphtheria toxin, allowing toxin-mediated selective depletion of wound macrophages. In each of these studies, complete toxin-mediated depletion of macrophages before the placement of wounds led to delayed wound closure, decreased granulation tissue formation and angiogenesis, decreased collagen synthesis, decreased numbers of myofibroblasts, and decreased levels of growth factors such as vascular endothelial growth factor (VEGF) and transforming growth factor-β (TGF-β). When macrophage depletion was limited to the tissue maturation phase, there was no significant effect on the healing outcome [7]. More recent studies of murine wounds show that removal of a single specific subset of mid-stage CD301b-expressing macrophages phenocopies the wound healing defects observed in mice where macrophages are completely depleted, thus narrowing the critical functional group [37]. Together, these many studies conclusively demonstrate that macrophages are essential to normal healing in adult rodents. A caveat to these studies is that most approaches deplete the entire macrophage population, leaving open the question of the specific roles of different macrophage subpopulations in wounds. Another consideration is that, despite complete or near complete depletion of macrophages, all of the wounds eventually healed. This outcome suggests that wound healing is adaptable and able to overcome even severe perturbations to achieve the necessary and life-preserving outcome of wound closure. 

## 3. Macrophage Function in Specific Wound Types

### 3.1. Fetal Wounds 

Contradictory evidence about the essential role of macrophages in wound healing has been documented in several systems, including early fetal wounds. Incisional skin wounds placed on fetal skin at early stages of development (within the first two trimesters or so) heal rapidly and in a scarless fashion. The inflammatory phase is greatly reduced in fetal skin wounds when compared to adult wound counterparts [12]. Differences that have been described include a near absence of neutrophils along with fewer mast cells that are more immature than those of adult skin [14,38]. Artificially increasing inflammation in scarless fetal wounds leads to scar formation, suggesting that the reduced inflammation is key to the remarkable healing outcome [39,40,41].

Concerning the role of macrophages in fetal wounds, macrophages are indeed present, although reduced in number and present for a shorter period than what is observed in adult wounds [12,40,42]. Moreover, some studies suggest that there are fewer activated macrophages in fetal wounds [42]. Aside from the somewhat circumstantial evidence that macrophages are dispensable to fetal repair, the role of macrophages in fetal wound healing has not been rigorously tested. The manner in which complete depletion of macrophages would influence healing in fetal wounds remains to be studied, leaving the door open for the possibility that macrophages might be needed in early-stage fetal repair. 

### 3.2. Mucosal Wounds 

The structure of the mucosa, as a barrier epithelial tissue, is quite similar to skin [43]. This similarity in the tissue architecture permits a direct comparison of healing in the skin and mucosa. Mucosal wounds, including those in the gingiva of the oral cavity and lining mucosa of the vagina, heal remarkably quickly and with reduced scar formation as compared to similar size skin wounds [44]. Many basic elements of repair are different in mucosal wounds, including reduced inflammation, accelerated re-epithelialization, and refined angiogenesis, reducing the need for capillary remodeling. The macrophage content in mucosal wounds is reduced in experimental models in both mouse and pigs, as well as in human wounds [45,46,47,48]. In mouse mucosal wounds, the macrophage content is most reduced early in healing, at a time where inflammatory macrophages predominate. At the later stages of healing, the concentration of macrophages in mucosal wounds nears that found in skin. While minimal phenotypic analysis is yet available, the stoichiometry suggests that rapid mucosal healing involves a decrease in inflammatory macrophages, with normal levels of reparative macrophages. This altered response may result from the known innate differences in the epithelial response to injury in the mucosa versus the skin. As compared to the skin epithelium, injured oral mucosal cells produced reduced levels of cytokines, a situation that contributes to reduced inflammation and may influence macrophage infiltration and phenotype [46,49,50]. The genomic pattern of healing in the skin and mucosa is remarkably different, as mucosal wounds express only half or so of the genetic elements that are expressed in skin wounds [49,51], and oral wounds exhibit distinct patterns of regulatory miRNAs [52]. In addition, levels of stem cells are higher in the mucosa than in the skin, a situation that may dampen the inflammatory response and support rapid tissue restoration [53].

The finding that both fetal and mucosal wounds heal well yet exhibit reduced macrophage content suggests that there may be a trade-off between a protective anti-microbial inflammatory response and a less inflammatory yet more regenerative response. The concept that inflammatory responses in healing wounds might be both friend and foe in terms of healing outcomes is not a new one, and much remains to be learned about the optimal inflammatory response in wounds [54,55]. In terms of macrophage function, studies to test just how much macrophage activity is needed to combat routine infection in skin wounds are few, leaving the matter of optimal macrophage response (i.e., one that supports healing without undue infection) an open question. While neither fetal nor oral wounds exhibit increased infection (as compared to skin), this situation could be explained by anatomical characteristics, including the in utero environment of the fetus and the anti-microbial activity of saliva in the oral cavity. 

## 4. Macrophage Function in Wounds in Specific Model Organisms

### 4.1. PU.1-Deficient Mice

The transcription factor PU.1 is an ETS family transcription factor that is responsible for the maturation and functional competence of several haemopoietic lineages. Mice that are rendered genetically deficient for PU.1 (i.e., PU.1-knockout mice) lack both macrophages and neutrophils. Remarkably, PU.1 -/- mice heal extremely well and repair skin wounds in a time course similar to normal mice [11]. In addition to having a similar time course of wound closure, PU.1 -/- mice exhibit improved repair, as they heal with no obvious scar formation. Other features of repair in PU.1 -/- wounds include decreased cell death and ingestion of dying cells by fibroblasts. A few limitations in this study are noted. First, the study was performed in neonatal rather than adult mice, and second, antibiotic therapy was employed following injury to reduce the chance of infection. These factors prevent a comparison of these findings to studies of adult immunocompetent mice. Nevertheless, this finding underscores the resiliency of the wound healing process and demonstrates that PU.1 mice are a compelling example of normal, or perhaps even improved, healing in the absence of macrophages. 

### 4.2. The Spiny Mouse

The spiny mouse is unique among mammals, as this species can fully generate skin and hairs following wounding. The skin of the spiny mouse, a species actually more closely related to hamsters than mice, is quite loose, allowing the species to escape and survive attacks by predators even when large sections of skin are removed [56]. A thorough comparison of the inflammatory response in the spiny mouse versus the lab mouse, Mus musculus, has been performed in 8-mm full thickness skin wounds [13]. Using F4/80, a widely used macrophage marker, wound analysis of Mus versus Acomys showed that while F4/80+ cells were present in Mus wounds in the expected pattern, no F4/80+ macrophages were seen in the Acomys wounds at any time point that was examined, even though F4/80+ cells were present in spleen of Acomys. While the data seems to show that Acomys heals well without macrophage involvement, a possibility remains that F4/80+ is not an ideal marker for dermal macrophages in Acomys. It may be that in this species, certain subpopulations of macrophages may not express this marker. 

## 5. Macrophages in Regenerative Species

Several species, most notably amphibians and fish, show remarkable regenerative capacity and can completely regenerate organs, skin, fins, and limbs [57]. These observations have captured the attention of many scientists, as they suggest the eventual possibility that humans might be effectively re-programmed to regenerate tissues, organs, and limbs after injury. Among regenerative species, amphibians are one of the most studied species due to their lifelong regenerative capacity. In particular, the salamander has been widely studied as it can regenerate multiple tissues and organs, and even limbs, repeatedly throughout life [58]. The regenerative capacity of amphibians derives from many factors but clearly relies upon a successful immune response. Indeed, limb regeneration in salamanders specifically depends upon appropriate and adequate macrophage function [59]. In the absence of macrophages, fibrous tissue production occurs in excess, shutting down the regenerative pathway. The role of macrophages in salamander regeneration, promoting regeneration while reducing fibrosis, contrasts directly with their ascribed role in mammalian wounds where macrophages are often considered to promote scar formation and fibrosis. For example, studies of mouse cardiac injury show that macrophages contribute to collagen formation, a situation that supports rapid repair to prevent cardiac rupture but can ultimately lead to scar-related dysfunction [60]. Excessive scarring and fibrosis have been linked to macrophage dysfunction in a wide range of injuries [61]. The topic of macrophages as critical mediators of scar formation has been widely studied and thoroughly reviewed recently [62]. In short, the balance between regeneration of tissue and scar-forming repair seems linked to macrophage function, with excessive inflammation leading to fibrotic rather than regenerative repair. 

## 6. Macrophage Function in Poorly Healing Wounds and Scars

Given the importance of macrophage function in normal wounds, it is no surprise that macrophage dysfunction has been documented to play a role in poorly healing wounds. Most notable are studies showing that macrophage dysfunction is prominent in the poorly healing and non-healing wounds that occur in the context of diabetes. Macrophage dysfunction in diabetes is multi-factorial and includes impaired ability to clear apoptotic cells, impaired growth factor production, and altered inflammatory activity [26,63,64]. The best studied of these deficits, however, is the failure of macrophages to adopt a pro-healing reparative phenotype, being generally stuck in a pro-inflammatory state [28]. The transition of macrophages to a reparative phenotype is regulated at an epigenetic level, of which the differential expression of the methyltransferase Setdb2 which occurs in diabetic wounds is a critical component [65]. Treatments that modify the macrophage phenotype, using either mechanical, pharmacologic, or biologic approaches, have been shown to improve and speed healing in both diabetic mice and humans [66,67,68,69]. Although no clinical treatments that focus on macrophage dysfunction are yet available in the clinic, this is a promising and exciting area of great translational importance. 

Macrophage dysfunction propagates a pro-inflammatory and tissue-destructive environment that impedes healing. Many developing therapeutic approaches attempt to switch pro-inflammatory cells towards a reparative phenotype. However, it is not clear whether the persistence of a large population of pro-inflammatory macrophages or a lack of reparative macrophages, or both, drives impaired healing. In the former case, the simple removal of dysfunctional macrophages might be sufficient to turn the tide and allow chronic wounds to heal. Removal of abnormal macrophages, which has not yet been explored, is quite difficult to study experimentally, yet future technologies may allow this question to be investigated. 

Though a deficit of macrophage function has been implicated in the pathology of chronic ulcers, some studies implicate excessive macrophage activity in the formation of hypertrophic scars. Macrophages are present in scars, and a higher number of M2 macrophages in uninjured skin has been associated with increased prevalence of hypertrophic scars following injury in humans [70,71]. In an athymic nude mouse model involving a transplanted human hypertrophic scar, the systemic depletion of macrophages has been shown to reduce scar formation [72]. In tandem with our understanding of chronic wounds, these studies demonstrate the importance of the correct balance of macrophage function to the appropriate healing response [73]. 

## 7. A New Paradigm for Macrophage Function in Wounds 

The range of macrophage functions and importance, as studied in wound models, spans the spectrum from completely dispensable to essential for repair. Here, we consider how these apparently disparate findings might be reconciled. 

### 7.1. Substitutional Cell Types in Wounds 

**Figure 1:**
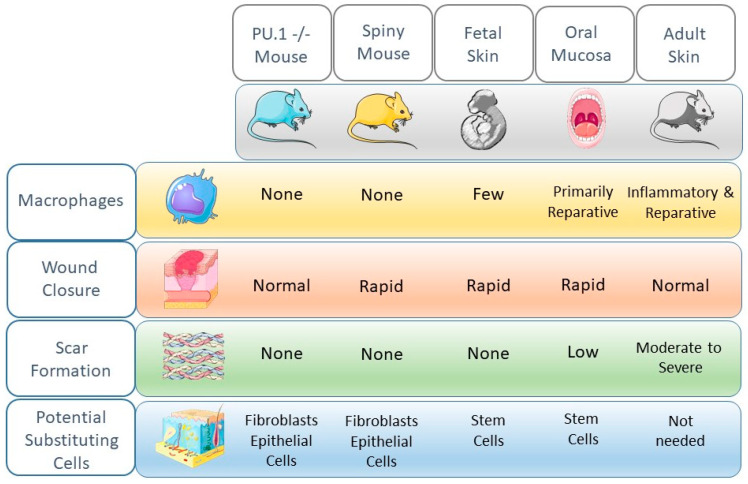
Comparison of healing phenotypes, macrophage content, and possible cells that might substitute for macrophages. Several model systems show that wounds can heal well in the absence of or with a reduction in the macrophage population. In many cases, the absence of macrophages might be compensated for by other cell types that can adopt new functions. Artwork is courtesy of Smart Servier Medical Art, freely available at https://smart.servier.com/.

In wounds that heal without macrophages, or with minimal macrophage function, one possibility may be that macrophage function in wounds is replaced by other cells (Figure 1). Evidence that other cell types might “pinch hit” for macrophages can be found in multiple systems and studies. Firstly, in terms of cytokine and growth factor production, many types of wounds exhibit production patterns that overlap with macrophages. When cells from wounds were sorted into neutrophils/T cells/B cells (NTB cell subset), monocytes/macrophages (Mo/Mp subset), and non-leukocyte cells including keratinocyte/fibroblast/endothelial cells (KFE subset), overlapping production of cytokines and growth factors was observed among these different cellular populations (Table 1) [74]. Although the macrophage population was a prominent producer of both inflammatory cytokines and growth factors, the NTB subset also produced pro-inflammatory cytokines, while the KFE subset produced significant levels of growth factors. In particular, keratinocytes are well described to be pro-inflammatory following injury and to be essential regulators of wound inflammation [75].

Regarding the phagocytic capacity of wound macrophages, the existing evidence allows us to safely assume that the clearance of apoptotic cells is essential to healing. In wounds that heal without macrophages, or with minimal macrophage function, this phagocytic function may be replaced by other cells. Evidence for such substitution of function comes from the study of wound healing in the PU.1 mouse [11]. Wounds in this mouse, which heal quickly and scarlessly in the absence of macrophages (also discussed above), contain high levels of phagocytic fibroblasts that may stand in for efferocytotic macrophages [11]. 

As far as tissue regrowth is concerned, redundancy in the production of growth factors by multiple cell types is well known [76]. For example, VEGF, a dominant proangiogenic factor in wounds, is produced not just by macrophages, but also by keratinocytes, fibroblasts, and endothelial cells themselves [77]. Additional compensation that may improve tissue regrowth probably comes in the form of stem cells. For example, in rapidly healing oral mucosal wounds, an abundance of neural crest-derived cells with stem cell-like capabilities has been suggested to quickly support cellular growth and restoration of tissue architecture, a function that might supplant the need for abundant macrophages [49,78]. In thinking about substitutional cells in wounds, an important consideration is cellular plasticity. Cellular plasticity has been demonstrated in wounds; for example, recent studies suggest that wound macrophages can sometimes convert to a fibroblast phenotype [79], and adipocytes have been shown capable of conversion to a fibroblast phenotype [37]. The extent of cellular plasticity in healing is not yet well understood, nor are the signals that might govern such events especially well described [80]. However, cell plasticity certainly supports the idea that cells might substitute for one another in wounds. Overall, then, the concept of substitution of function provides a reasonable explanation for the ability of certain wounds to heal quite well with a relative sparsity of macrophages. 

### 7.2. Proportionality of Macrophage Phenotypes 

**Figure 2:**
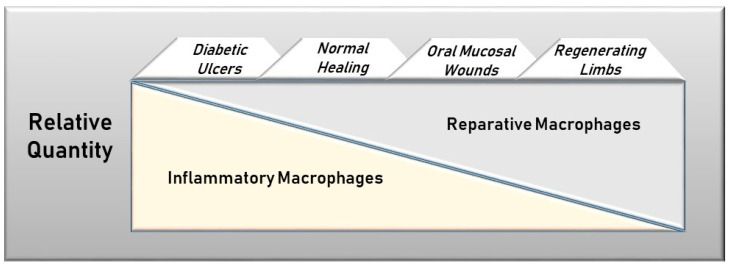
Relative levels of inflammatory versus reparative macrophages in differentially healing wounds. The healing outcome may depend upon the proportion of inflammatory versus reparative macrophages in the wound. In non-healing diabetic ulcers, inflammatory macrophages are predominant, while only reparative macrophages are found in regenerative limbs of salamanders. Oral mucosal wounds have both types of macrophages, but the reparative phenotype dominates.

When macrophages are abundant in wounds, the relative proportion of inflammatory to reparative macrophages is likely to be an important rheostat of healing outcomes (Figure 2). At one end of the spectrum is the regenerating salamander limb with an entirely regenerative/reparative macrophage population and at the other end is the diabetic ulcer with an abundance of inflammatory macrophages that are resistant to a phenotypic switch. The phenotypic proportion, therefore, might dictate the ultimate healing trajectory. In keeping with this concept, the addition of large numbers of reparative macrophages has been shown to reduce the healing deficit in diabetes and in aging, both circumstances where inflammatory macrophages dominate [81,82,83]. Taken further, this model might logically lead to the idea that even in normal wounds, a selective decrease in inflammatory macrophages, if accomplished without significant impairment of reparative macrophages, could improve healing outcomes. To be sure, several studies do suggest that some level of inflammatory macrophage activity is beneficial to repair [63,84,85]. Nevertheless, a reduction in inflammatory macrophages might diminish the bystander tissue destruction caused by early inflammation. As a result, the wound would exhibit rapid repair of a smaller injury. In support of this idea, macrophages in the regenerating limbs of salamanders are non-inflammatory and growth-promoting [59]. In sum, the data strongly suggest that the relative characteristics of macrophages dictate healing outcomes.

### 7.3. Macrophages as Negative Regulators of Inflammation

If macrophage function in wounds can be supplanted by other cell types, how might we explain the many sophisticated studies that show that complete macrophage depletion results in aberrant and slow healing? One possibility is that complete removal of macrophages sets up an excessive inflammatory response that remains in play longer than normal. This inflammation would lead to tissue destruction beyond that of a normal wound, thus lengthening the time needed to repair the site. Partial support for this concept comes from studies of wound healing in macrophage-depleted mice, where the inflammatory mediator TNF-alpha is increased in the later stages of repair [6]. Macrophages can indeed dampen inflammation, and a subset of tissue-resident macrophages located in perivascular regions of the skin have been found to express an anti-inflammatory transcriptional profile as well as assisting in tissue remodeling [86]. In normally healing wounds, the anti-inflammatory/remodeling subset of macrophages may be most essential to repair, along with small numbers of reparative macrophages. In truth, then, the number of macrophages that are required in adult skin for appropriate wound healing may be much less than what appears in normally healing wounds. As intriguing as such a concept is, experimental approaches are limited. Precise titration of macrophages and, perhaps more importantly, rigorous control of in vivo phenotypes are experimentally quite challenging in wounds. 

## 8. Remaining Questions and Future Directions

A detailed picture of the role of macrophages in wounds is emerging, with nearly 4000 manuscripts now published on this topic. The accumulated literature unequivocally demonstrates that macrophages have multiple functions in wounds and that aberrant macrophage functionality is a common feature of poorly healing and non-healing wounds. Perhaps not surprisingly, macrophage-based wound therapeutics have continued to receive experimental attention, although no commercial macrophage therapeutics are yet available (for a review, see Spiller and Koh, 2017 [87], Krzyszczyk et al., 2018 [88], and Snyder et al., 2016 [89]). The development of effective macrophage therapeutics requires continued study, and several important and thorny questions remain. More investigations are needed to delineate the regulation of macrophage phenotypes in wounds, as this information can inform the design of appropriate therapies for both non-healing wounds and macrophage-dependent fibrotic responses. A complete understanding of the complexity of macrophage function in wounds will demand advanced analysis, including single-cell phenotypic analysis. As technology progresses, advanced computational approaches will be needed to make sense of the large datasets that are generated. Finally, additional studies of functional cellular substitution and cellular plasticity of cells within the wound will be needed. These and many future studies will advance our comprehension of the apparent paradoxes concerning the role of macrophages in the repair response, with the potential of identifying novel therapeutics to improve clinical healing outcomes.

## 9. Methods

A PubMed search was performed for the terms “macrophages” and “wound healing” for the period of January 1900 to June 2020. This resulted in 3798 citations. Manuscripts were reviewed for relevance to the topic of this review, as well as for citations related to the topic of the review. Approximately 350 manuscripts were found to be relevant to the topic of the review and examined for inclusion. This review should be considered a narrative rather than a systematic review. 

## Figures and Tables

**Table 1:** Overlapping production of cytokines and growth factors by different wound cell types at day 5 post-injury (derived from Mirza and Koh, 2016).

	Mediator						
Cell Type	IL-1β	TNF-α	IL-6	IL-10	VEGF	IGF-1	TGF-β
Macrophages	+++	+++	++	+	++	+	+
Neutrophils/T cells/B cells (NTB)	+	+++	+	+	-	+	+
Keratinocytes/fibroblasts/endothelial cells (KFE)	+	-	+	+	+++	++	+

## Data Availability

Not applicable.
